# Gastric Adenomyoma: A Rare Entity Mimicking Pyloric Neoplasia—A Case Report

**DOI:** 10.1155/carm/9964542

**Published:** 2025-09-22

**Authors:** Valentina Velasco-Muñoz, J. Santiago Pabón-Castro, María Camila Escudero-Bohórquez, Andres G. Aponte-Vargas, Gabriel Alberto Roa-Rossi, Jose Manuel Sánchez Jaramillo, Pablo García-Echeverri, William H. Salamanca-Chaparro

**Affiliations:** ^1^Medical School, Pontificia Universidad Javeriana, Bogotá, Colombia; ^2^Surgery Department, E. S. E Hospital Universitario de La Samaritana, Bogotá, Colombia; ^3^Medical School, Fundación Universitaria Sanitas, Bogotá, Colombia; ^4^Surgery Department, Universidad de La Sabana, Bogotá, Colombia

**Keywords:** case report, gastric adenomyoma, gastroduodenostomy, pyloric lesion

## Abstract

**Introduction:** Gastric adenomyoma is a rare benign lesion that may present with nonspecific gastrointestinal symptoms or be incidentally discovered. Due to its rarity and imaging resemblance to malignant tumors, it often leads to diagnostic and therapeutic dilemmas.

**Methods:** A retrospective study was conducted, and a literature review was made to describe the background of the case found.

**Case Report:** We report a case of a 50-year-old female who developed a gastric adenomyoma mimicking a pyloric lesion initially suspected to be malignant. Histopathological examination confirmed the diagnosis of gastric adenomyoma. The patient's postoperative course was uneventful, and she remains asymptomatic on follow-up.

**Conclusions:** Gastric adenomyoma should be considered in the differential diagnosis of pyloric masses. Awareness of its imaging and histopathological characteristics can prevent unnecessary radical surgery.

## 1. Introduction

Gastric adenomyoma is a rare benign tumor-like lesion characterized by glandular structures embedded within smooth muscle layers of the stomach. First described by Magnus-Alsleben in 1903, this entity remains extremely rare, with limited cases reported in the literature. Histologically, adenomyomas are composed of proliferating ducts and glands lined by columnar epithelium, intermixed with smooth muscle bundles, often leading to misinterpretation as malignancies such as gastric adenocarcinoma or gastrointestinal stromal tumors (GISTs) [1]. The pathogenesis of gastric adenomyoma is not well understood, but several hypotheses suggest that it may result from either embryologic developmental anomalies or constant hyperplasia secondary to chronic irritation. These lesions predominantly occur in the antrum and pyloric region, with the potential to cause significant gastrointestinal obstruction when they reach a critical size [2]. Due to its nonspecific clinical presentation and radiological overlap with malignant gastric masses, gastric adenomyoma poses a diagnostic challenge and often requires histopathological confirmation following surgical resection [3].

Despite being a benign entity, gastric adenomyoma can present with severe clinical manifestations, particularly when it results in pyloric stenosis. In such cases, patients may exhibit symptoms including chronic nausea, vomiting, epigastric pain, postprandial fullness, and progressive weight loss due to diminished gastric emptying [3]. These symptoms overlap with malignant gastric tumors which often lead to an extensive and sometimes invasive diagnostic workup. Conventional imaging studies, such as contrast-enhanced computed tomography (CT) scans and endoscopic ultrasonography (EUS), may reveal a submucosal mass, but these findings alone are insufficient for definitive diagnosis. Similarly, superficial endoscopic biopsies often demonstrate inconclusive or nonspecific results, as seen in this case, where initial histological reports indicated chronic gastritis without dysplastic or neoplastic changes [4]. This highlights the limitations of routine biopsy techniques in diagnosing deep submucosal lesions, reinforcing the need for additional diagnostic approaches such as recurrent biopsy techniques or surgical resection when malignancy cannot be ruled out [5].

This case report describes a 50-year-old female patient with progressive pyloric stenosis, refractory gastrointestinal symptoms, and significant weight loss over 2 months, initially suspected of harboring a malignant gastric tumor. Despite multiple endoscopic biopsies failing to demonstrate malignancy, persistent symptoms and imaging findings warranted surgical intervention. The final histopathological analysis of the resected specimen confirmed the diagnosis of gastric adenomyoma, a rare etiology of gastric outlet obstruction. This case emphasizes the diagnostic challenges associated with submucosal gastric lesions and emphasizes the importance of a systematic approach involving repeat biopsies, interdisciplinary discussion, and surgical assessment to achieve an accurate diagnosis. Of the few cases reported, most occur in the gallbladder, but some occur in the stomach antrum and pylorus [6]. By contributing to the limited existing literature, this report highlights the need for increased awareness of gastric adenomyoma as a differential diagnosis in patients presenting with gastric outlet obstruction and suspected malignancy, ultimately facilitating better clinical decision-making and optimizing patient outcomes.

## 2. Methods

**Figure 1:**
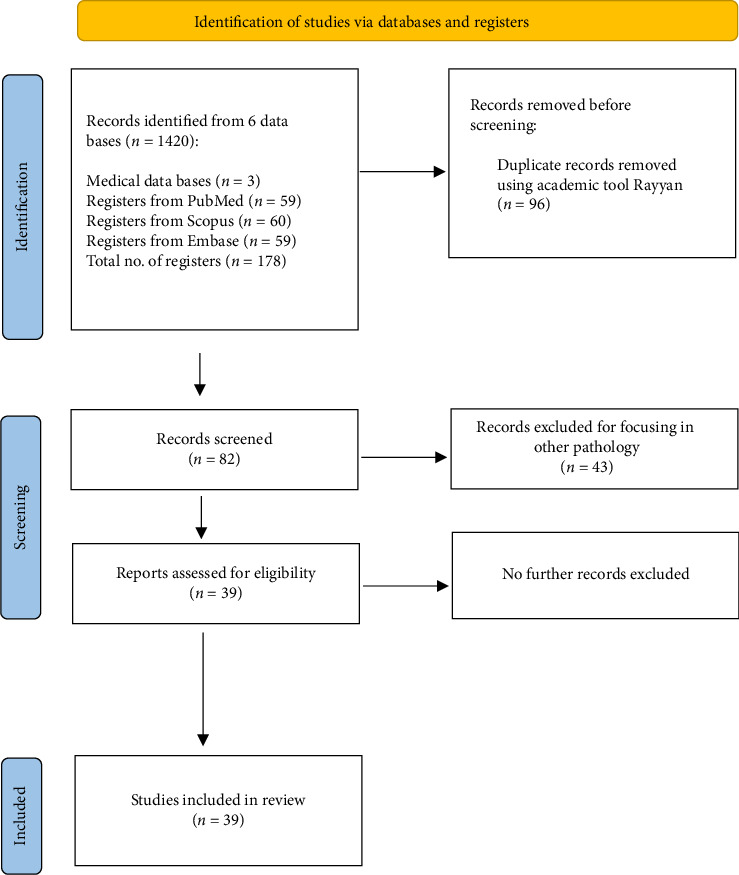
Identification of studies researching literature information about gastric adenomyoma report cases across PubMed, Scopus, and Embase.

A retrospective study was conducted using clinical information about a patient from E.S.E Hospital Universitario de la Samaritana, an academic and university hospital, following all due process counting with the ethical committee approbation. Additionally, a literature review was conducted to describe the background of the case found, as we illustrated in Figure 1.

The articles were read to better understand the case and clinical context of the type of pathology reported. As shown, we researched 3 major databases and only found 39 cases reported, which makes this case a valious contribution to the literature.

This research revealed several consistent clinical and pathological features. Most lesions occur in the pyloric and antral regions of the stomach, though rare instances have been described in other sites such as the gastric body [1, 3, 6]. The clinical presentation is often nonspecific, with epigastric pain, postprandial fullness, nausea, vomiting, and weight loss being common symptoms—particularly when the lesion leads to gastric outlet obstruction [1, 3, 4]. These features can mimic malignancy, complicating diagnosis. Imaging studies typically reveal submucosal masses, but they are insufficient to differentiate benign from malignant lesions, and superficial biopsies frequently return inconclusive results [3–5]. Histologically, gastric adenomyomas are characterized by benign ductal and glandular structures interspersed within smooth muscle fibers, which can resemble invasive adenocarcinoma or GIST, further complicating diagnosis [1–4]. Surgical resection is often performed due to suspicion of malignancy, with antrectomy or subtotal gastrectomy being the most common procedures [1, 3]. Most reported postoperative courses are uneventful, although a few cases describe minor complications such as delayed gastric emptying or infection, underscoring the importance of accurate diagnosis and tailored surgical planning [1, 3].

## 3. Case Presentation

**Figure 2:**
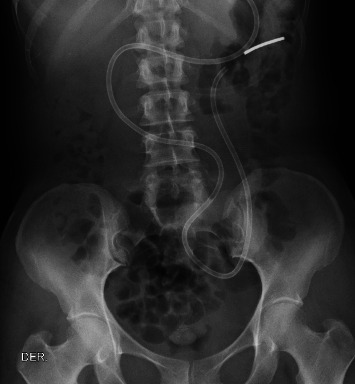
Supine abdominal radiograph showing the course of a nasoenteric tube reaching into the small bowel. Multiple mildly dilated small bowel loops with air-fluid levels are seen, consistent with a proximal obstruction pattern.

A 50-year-old female patient with a history of irritable bowel syndrome was admitted to the emergency department with a 3-week history of colic-type abdominal pain associated with multiple emetic episodes of fecaloid characteristics and significant involuntary weight loss (13 kg in 2 months). Upon admission, an abdominal X-ray was performed as shown in Figure 2, showing the presence of abundant fecal matter without signs of obstruction.

**Figure 3:**
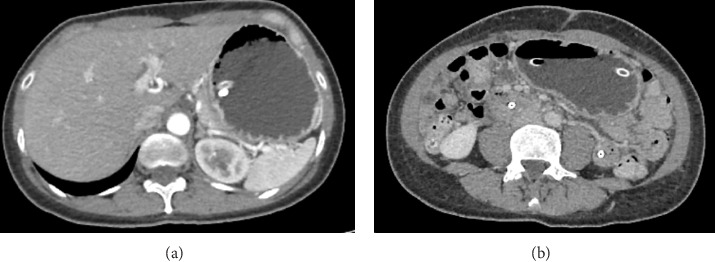
(a) Axial contrast-enhanced CT image showing marked gastric distension with an abrupt transition at the pyloric region. The gastric outflow tract narrows sharply into a collapsed bowel segment, consistent with gastric outlet obstruction. (b) Axial contrast-enhanced CT image of the mid-abdomen, demonstrating prominent gastric distension and multiple collapsed small bowel loops throughout the abdomen, consistent with a gastric outlet obstruction pattern.

However, given the constitutional syndrome and persistence of the symptoms, a CT scan of the abdomen with contrast was indicated, as shown in Figures 3(a) and 3(b), which showed marked dilation of the gastric chamber with an abrupt change toward the pyloric region with collapse of small intestinal loops.

**Figure 4:**
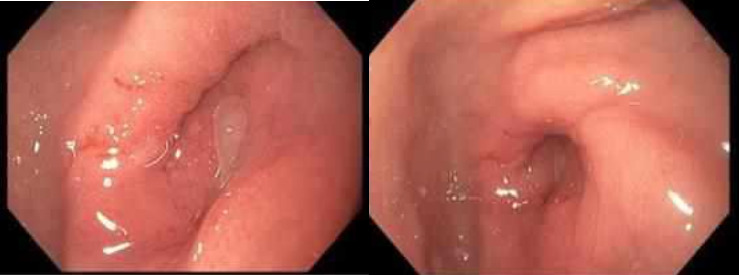
Upper digestive tract endoscopy (EGD) was performed. In the antrum, an eccentric pylorus was identified, associated with mucosal thickening and secondary stenosis. The mucosa showed edema and loss of normal pattern, raising suspicion for an infiltrative lesion at that level.

**Figure 5:**
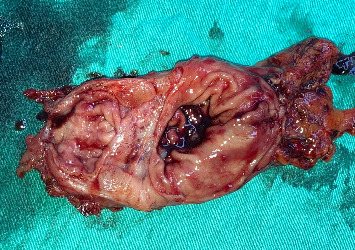
Gross pathological specimen of a resected gastric segment showing a submucosal lesion in the pyloric region. The surgical specimen, obtained from a subtotal gastrectomy with Roux-en-Y reconstruction, reveals areas of congestion, and hemorrhagic changes are visible, potentially due to chronic irritation. Histopathological analysis confirmed the diagnosis of gastric adenomyoma, a rare benign lesion composed of glandular structures embedded within smooth muscle layers, which contributed to the patient's gastric outlet obstruction.

Therefore, the patient is now treated with pyloric syndrome due to stenosis of etiology to be established, however with a high probability of malignancy. An upper digestive tract endoscopy (EGD) was performed as documented in Figure 4 with a biopsy of the pylorus as shown in Figure 5, which showed moderate inactive chronic gastritis, without signs of atrophy, dysplasia, or intestinal metaplasia, negative for malignancy. However, given the persistence of the condition, an interdisciplinary meeting was held where a new EGD was indicated to perform a biopsy-on-biopsy procedure to assess deep digestive tract involvement, which showed a gastric pseudodiverticulum as the etiology of the condition, with an indication for subtotal gastrectomy (antrectomy) with Roux-en-Y gastric bypass. A procedure was performed that showed the presence of mesenteric nodes at the root of the mesentery increased in size and number, however without the presence of carcinomatosis. In front of the gastric component, a submucosal mass was visualized at the level of the antrum and pylorus, approximately 2 cm in length, representing a significant circumferential involvement, which was observed at the time of opening the surgical piece, respecting the mucous layer. The procedure was performed without complications. Prior to discharge, a methylene blue test was performed on the gastroenteric and jejunal anastomosis with adequate passage, permeable and negative for leaks. Samples were sent labeled as “antrectomy product,” “mesenteric lymph node,” and “omental sample,” where the gastric sample showed morphological findings compatible with gastric adenomyoma.

Following surgery, the patient was admitted to the intensive care unit (ICU) for postoperative clinical surveillance and monitoring, given the high risk of hemodynamic complications. After four days of continuous monitoring with favorable clinical progression, transfer to the general ward was indicated. The patient subsequently completed a total of one week of observation and was discharged with appropriate recommendations and instructions regarding warning signs.

## 4. Discussion

Gastric adenomyoma is a rare benign lesion conformed by glandular and smooth muscle components, most commonly found in the antrum and pyloric region. Although typically asymptomatic, larger lesions can lead to gastric obstruction, mimicking malignant gastric tumors clinically and radiologically speaking. Due to its submucosal location, conventional endoscopic biopsies often fail to provide a definitive diagnosis, necessitating repeat biopsies or surgical resection for histopathological confirmation. This diagnostic challenge underscores the importance of considering gastric adenomyoma in patients presenting with progressive pyloric stenosis and constitutional symptoms. In this case, a 50-year-old female presented with colicky abdominal pain, fecaloid vomit, and significant involuntary weight loss. Initial imaging suggested gastric dilation with pyloric stenosis, raising suspicion of malignancy. Endoscopic biopsies were not conclusive, prompting a biopsy-on-biopsy technique, which revealed a gastric pseudodiverticulum. Given the persistence of symptoms, the patient underwent subtotal gastrectomy with Roux-en-Y reconstruction, during which a 2-cm submucosal mass at the pylorus was identified. Histopathological examination confirmed gastric adenomyoma, explaining the patient's symptoms. Postoperatively, the patient required ICU monitoring for hemodynamic surveillance but recovered well and was discharged after a week of observation.
